# Accurate estimation of a phase diagram from a single STM image

**DOI:** 10.1038/s41598-018-25283-1

**Published:** 2018-05-01

**Authors:** Kazuhito Takeuchi, Koretaka Yuge, Shinya Tabata, Hiroki Saito, Shu Kurokawa, Akira Sakai

**Affiliations:** Department of Materials Science and Engineering, Kyoto University, Sakyo, Kyoto, 606-8501 Japan

## Abstract

We propose a new approach to constructing a phase diagram using the effective Hamiltonian derived only from a single real-space image produced by scanning tunneling microscopy (STM). Currently, there have been two main methods to construct phase diagrams in material science: *ab initio* calculations and CALPHAD with thermodynamic information obtained by experiments and/or theoretical calculations. Although the two methods have successfully revealed a number of unsettled phase diagrams, their results sometimes contradicted when it is difficult to construct an appropriate Hamiltonian that captures the characteristics of materials, e.g., for a system consisting of multiple-scale objects whose interactions are essential to the system’s characteristics. Meanwhile, the advantage of our approach over existing methods is that it can directly and uniquely determine the effective Hamiltonian without any thermodynamic information. The validity of our approach is demonstrated through an Mg–Zn–Y long-period stacking-ordered structure, which is a challenging system for existing methods, leading to contradictory results. Our result successfully reproduces the ordering tendency seen in STM images that previous theoretical study failed to reproduce and clarifies its previously unknown phase diagram. Thus, our approach can be used to clear up contradictions shown by existing methods.

## Introduction

Exploring new high-functional materials has remained a greatly challenging theme for several decades. Phase diagrams play very important roles in their manufacture. To obtain phase diagrams, both experiments and simulations have to be used. One can roughly classify the methodologies based on simulations into two main types: one is CALPHAD^1,2^ using thermodynamic information obtained by experiments and/or other simulation methods, and the other is *ab initio* calculations without experiments. In particular, for estimating alloy phase diagrams based on microscopic information, *ab initio* calculations based on density functional theory (DFT)^3–7^ are now most widely used to determinie the interactions of a given system. DFT has been applied to bulk, surface, and interface systems and has achieved a number of successful results^8–10^.

**Figure 1:**
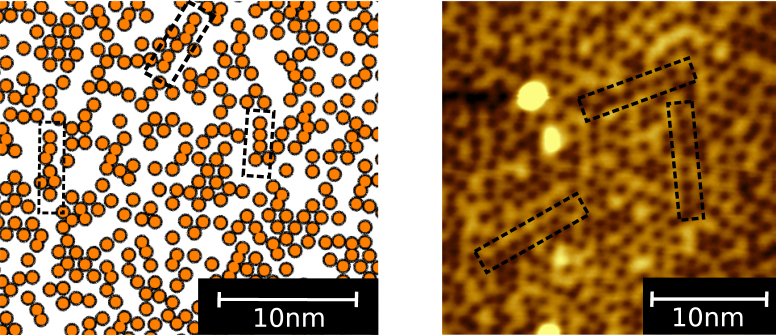
(Left) A snapshot of a Monte Carlo simulation at *x* = 0.0511 and *T* = 773 K. For clarity, only the Zn–Y clusters are shown. (Right) A typical STM image of the distribution of Zn–Y clusters in the stacking fault interface. Individual Zn–Y clusters are imaged as dark spots under positive bias voltage (empty-state image). In both images, chain-like arrangements of clusters are denoted by broken-line squares.

Meanwhile, there remain challenging systems where the results of *ab initio* calculations contradict with those of experiments. Such contradictions can particularly occur in systems where long-range and/or many-body interactions between multiple-scale objects (e.g., atoms and coarse-grained particles) are important because it is typically difficult to determine an effective multiscale Hamiltonian that appropriately describes the behavior of structural phase transitions. For DFT, it can be practically unfeasible to obtain an accurate Hamiltonian, especially when long-range interactions on a coarse-grained scale come into play, mainly due to the restricted size of the cells used; for experiments, essential difficulties arise in dividing thermodynamic information into individual multiscale interactions. One representative system, that includes such contradictions is Mg-based long-period stacking-ordered structure (LPSO), which has recently attracted a great deal of attention due to its considerable mechanical performance^11–13^. At the stacking-fault in LPSO, so-called nano-sized “clusters” consisting of multiple elements are arranged in Mg solvent, and this interface plays a central role in stabilizing the whole LPSO. Through *ab initio* calculations and Monte Carlo simulation, Kimizuka *et al*.^14^ determined the multiscale Hamiltonian with pair-wise intercluster interaction energies and revealed that the in-plane nanocluster-ordering tendency is mainly due to the repulsive interaction between nanoclusters. Although they partially explained the radial distribution function of clusters, the microscopic ordering tendency seen in STM images, i.e., chain-like cluster ordering shown in Fig. 1(b), was not quantitatively addressed. Minamoto *et al*.^15^ also constructed ternary bulk Mg–Zn–Y phase diagram using CALPHAD. However, since it is difficult to measure the thermodynamic information of a multiscale system, experiments to determine its Hamiltonian are hard to perform. Moreover, CALPHAD cannot show geometrical information concerning the ordering of nanoclusters.

In this study, we present a new approach to constructing a phase diagram by combining our recently proposed theory^16,17^ with two-dimensional STM images. Since our approach directly determines underlying many-body interactions only from the geometrical information of any-sized objects at thermodynamic equilibrium within a given accuracy, we can clarify that underlying interactions play an essential role in stabilizing the measured microscopic structure. Thus, when contradictions occur between *ab initio* calculations and experiments, it is expected that our approach, which does not require any thermodynamic information, can bridge this gap. To demonstrate the validity of our approach, the stacking-fault interface of Mg–Zn–Y alloy, in which L1_2_-type Zn_6_Y_8_ clusters are arranged, is a suitable target. We herein show the interface phase diagram of the 18R-type LPSO phases in Mg–Zn–Y alloy, compare our results with previous studies, and successfully reproduce the short-range-order (SRO) of clusters seen in the STM image, which the previous work failed to reproduce quantitatively.

## Results and Discussion

On the right-hand side of Fig. 1, we show a typical STM image of the distribution of Zn–Y clusters in the stacking-fault interface (see Methods for sample preparation). Since the STM is only sensitive to the topmost surface atoms, one can directly determine the individual positions of the clusters^18^. Before the evaluation of the SRO, we corrected the distortion of STM images, which originates from thermal drift. In particular, we calculated the vectors between all clusters and selected those corresponding to the 6th-nearest-neighbor (6NN) distance. STM images were corrected by linear transformation such that 6NN vectors had directions and magnitudes that were as correct as possible. Once we eliminated the effects of thermal drift, we could measure the relative positions between any pairs of clusters. We counted the number of cluster–cluster, cluster–vacancy, and vacancy–vacancy pairs in the field of the STM image up to a distance of 25NN and evaluated SRO.

**Figure 2:**
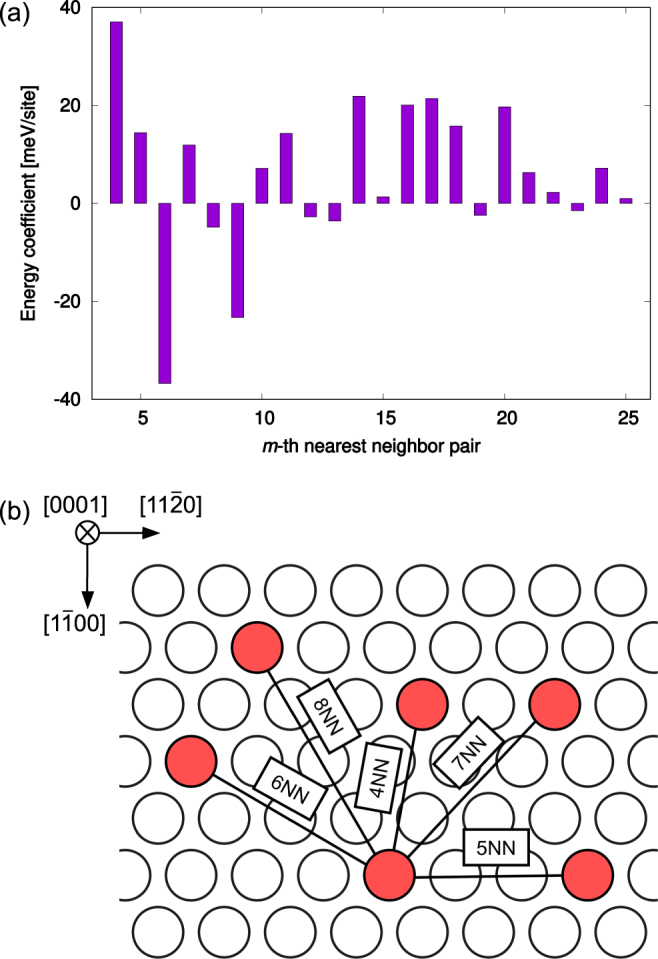
(**a**) Energy coefficients estimated by geometrical information, i.e., the SROs of Zn–Y clusters evaluated from a single STM image on the right-hand side of Fig. 1. (**b**) Schematic illustration of *m*NN pairs (*m* = 4–8) at the interface.

Energy coefficients of *m*NN pairs in Eq. (2), *V*_*m*_, as determined by SRO from STM images (see Methods) are shown in Fig. 2(a). We also shows a schematic illustration of some *m*NN pairs at the interface in Fig. 2(b). Since in 18R-type LPSO, the interplane interactions are relatively weak^19^, this method is valid for reproducing the characteristics of an STM image, ignoring interplane interactions and regarding the interface as an isolated two-dimensional system, i.e., all interactions in Fig. 2 are in-plane interactions.

In order to confirm validity of *V*_*m*_ in Fig. 2(a), we investigated the pair-formation energy of a single *m*NN (*m* = 6, 7, 8) cluster–cluster pair with an *L* × *L* two-dimensional triangular lattice under a periodic boundary condition. The pair-formation energy of Δ*E*(*m*, *L*) is defined as

1 $${\rm{\Delta }}E(m,L)=E(m,L)+{E}_{{\rm{Mg}}}-2{E}_{{\rm{cluster}}}(L).$$

**Figure 3:**
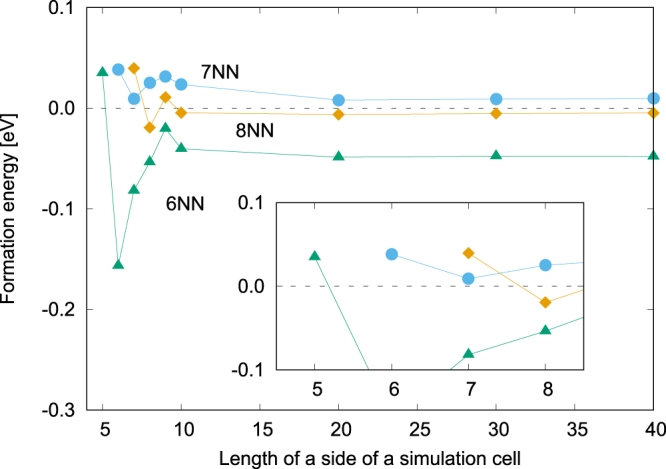
Pair-formation energy of a single *m*NN cluster–cluster pair, as defined by Eq. (1), for each *L*×*L* two-dimensional triangular lattice. The inset emphasizes the behavior of pair-formation energy in *L* = 5–8 simulation cells.

Here, *E*_Mg_ denotes the total energy of a configuration filled with Mg, and *E*_cluster_ denotes total energy of a configuration consisting of a single cluster and Mg atoms on the rest lattice points in a considered cell. The definition of Eq. (1) corresponds with pair interactions used in the previous DFT work^14^ and reflects actual *m*NN cluster-cluster pair interactions. In Fig. 3, Δ*E*(*m*, *L*) is shown for *m* = 6–8 and *L* = 5–40.

Figure 3 shows that positive values of Δ*E*(6, 5), Δ*E*(7, 6) and Δ*E*(8, 7) imply that 6–8NN cluster–cluster interactions behave repulsively under very small simulation cells, i.e., very dense cluster compositions. This tendency is similar to the result obtained by the previous DFT work^14^, which estimated interactions using a simulation cell with fully arranged clusters. Meanwhile, under dilute cluster compositions where *L* ≥ 10, Δ*E*(6, *L*) becomes strongly negative unlike Δ*E*(7, *L*) or Δ*E*(8, *L*). Negative value of Δ*E*(6, 40) in Fig. 3, which means 6NN cluster–cluster pairs are favorable, seems to contradict with the previous DFT result^14^, which held that 6NN cluster–cluster pairs are unfavorable. However, since strong oscillation of Δ*E*(*m*, *L*) in *L* ≤ 10 means that cluster–cluster interactions depend on the cluster composition, we actually clarify that the most probable reason for this disagreement is originate from insufficient consideration of dependence on cluster compositions in the previous DFT work^14^. Therefore, the 6NN cluster–cluster interaction behaves attractively for dilute clusters.

**Figure 4:**
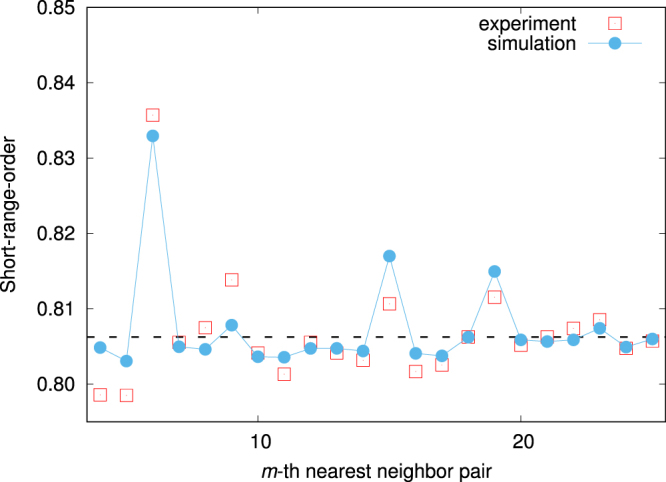
The SRO parameter of Mg_0.9489_(Zn_6_Y_8_)_0.0511_ for each *m*th-nearest-neighbor pair at *T* = 773 K. The broken line denotes the statistical average. If SRO is higher than average, the number of Mg–Mg and cluster–cluster pairs is larger than the number of Mg–cluster pairs. The opposite is true if SRO is lower than average.

Although the previous result^14^ successfully showed qualitative landscapes of the radial distribution function of nanoclusters, it could not reproduce the quantitative microscopic ordering tendency seen in STM images (solid lines in Fig. 1). This is because the previous DFT work determined cluster–cluster interactions by dense-cluster simulation cells, whereas we found that interactions behave differently between dense and dilute cluster concentrations, as shown in Fig. 3. Now, let us confirm that our multiscale interactions in Fig. 2(a) can capture the characteristics of SRO of clusters at the interface. Figure 4 shows thermodynamically averaged SRO at equilibrium, as obtained by Monte Carlo simulation (see Methods for a more detailed procedure), and this SRO corresponds to the radial distribution function of clusters. The strong SRO of 6NN pair means that the number of Mg–Mg and cluster–cluster pairs are relatively larger than that of Mg–cluster pairs, i.e., clusters are arranged so as to be at each other’s 6NN position. Our SRO landscape agrees well with the radial distribution functions obtained by previous DFT and experimental work, especially for the peaks in 6, 9, and 15NN pairs. For further discussion, we took a snapshot at the interface in the simulation and compared it with an STM image in Fig. 1. From the STM image, we can clearly confirm chain-like cluster ordering, unlike the previous DFT work, which showed a uniform arrangement of clusters. As above, our snapshot quantitatively captures the features of STM images and supports the validity of our multiscale interactions.

**Figure 5:**
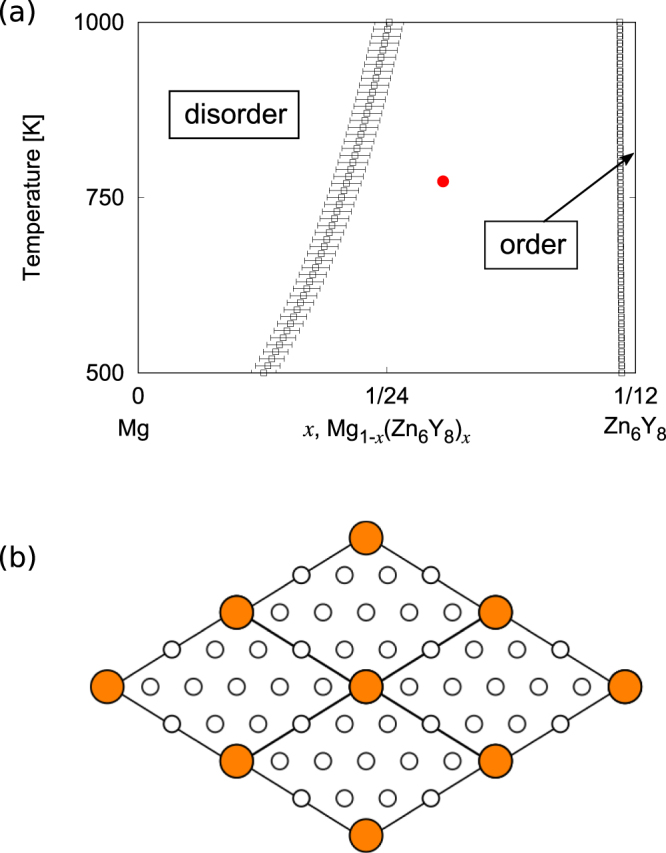
(**a**) ${{\rm{Mg}}}_{1-x}{({{\rm{Zn}}}_{6}{{\rm{Y}}}_{8})}_{x}$ interface phase diagram. Black lines denote the phase-coexistent line between the ordered and disordered phases with error bars. Closed circle denotes a point where the STM image in Fig. 1(b) is obtained. (**b**) Ordered phase of the interface. Large and small circles denote Zn–Y clusters and Mg, respectively.

The Mg_1−*x*_(Zn_6_Y_8_)_*x*_ interface phase diagram obtained by different three STM images is presented in Fig. 5(a), which shows the ordering tendency in the cluster-rich phase (Fig. 5(b)) through a first-order order-disorder phase transition. Since, the interplane interactions are relatively weak in 18R-type LPSO^19^, it is valid to regard the interface of LPSO as an isolated two-dimensional system. As a result, the closed circle in Fig. 5(a), showing the point where the STM image in Fig. 1(b) is obtained, indicates that Fig. 1(b) is not a single phase but a order-disorder phase coexistence state.

In summary, we suggested a new approach based on microscopic geometric information for constructing phase diagrams in cases where this is difficult for conventional simulation methods. Using STM images, we demonstrated our approach through the interface of 18R-type Mg–Y–Zn LPSO. We clarified the contradictory information concerning cluster arrangements presented by experiments and simulations and successfully reproduced an interface phase diagram consistent with that obtained by experiment. Our approach is expected to become a powerful tool for modeling isolated systems subjected to experimental observations such as STM.

## Methods

### Sample preparation for STM images

The directional solidification process was applied to a Mg_85_Zn_6_Y_9_ ingot. The ingot was annealed at 773 K for 168 h and quenched with water. We confirmed the presence of a diffraction spot corresponding to 18H-type LPSO by TEM observation.

Samples for STM observation were prepared by cutting the alloy ingot, typically into a reed shape of 8 mm × 5 mm × 0.5 mm. After the introduction of the sample into the preparation chamber of the low-temperature ultrahigh-vacuum STM (Unisoku 1200), we cooled the sample with a liquid-nitrogen flow for 10–15 min. Finally, we cleaved the sample using a pushing rod in the UHV chamber. The cleaved sample was immediately transferred into a pre-cooled observation chamber. All STM observations were carried out at liquid-nitrogen temperature (up to 77 K).

**Figure 6:**
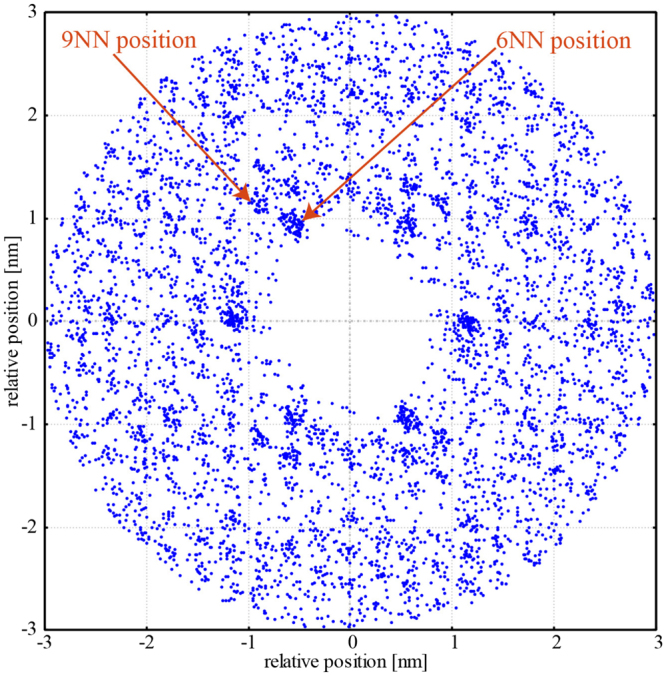
Relative positions of the clusters measured from all the clusters in a single STM image in Fig. 1 (Right).

STM itself has enough spatial resolution to resolve each atomic position on the surface. However, due to some noise and thermal drift, the positions of the clusters determined by our method have some uncertainty. To demonstrate the accuracy of our method, we plotted relative positions of the clusters measured from all the clusters in a single STM image in Fig. 6. As clearly seen, the relative positions of the clusters construct a lattice; which indicate that the accuracy of positions is good enough to estimate SRO.

### Generalized Ising model

In recent alloy studies with *ab initio* calculations, the generalized Ising model^20^ (GIM) has often been used for constructing a coarse-grained Hamiltonian, which captures the characteristics of a system within a given accuracy. Since we are interested in how clusters would be arranged at the interface with Mg solvent, for simplicity, it is natural to consider the clusters as coarse-grained atoms, i.e., Mg and the center of cluster as +1 and −1 Ising-spin variables, respectively. In GIM, the total energy of a given configuration, *σ*, is expanded by a set of complete and orthogonal basis functions, $\{{\xi }_{k}\}$, with indices

2 $${E}^{(\sigma )}=\sum _{k}{V}_{k}{\xi }_{k}^{(\sigma )},$$

where *V*_*k*_ denotes an energy coefficient and *k* is an index specified as a symmetrically nonequivalent figure such as 1NN and 2NN pairs. In particular for a binary system, *ξ*_*k*_ corresponds to the average product of spin variables *s*_*α*_ on *k* over all symmetrically equivalent *k* in an Ising-spin configuration

3 $${\xi }_{k}^{(\sigma )}=\frac{1}{{N}_{k}}\sum _{k\in \sigma }\prod _{\alpha \in k}{s}_{\alpha },$$

where *α* denotes a site on a given lattice and *N*_*k*_ denotes the number of *k* in the configuration.

*V*_*k*_ is defined as the inner product between total energy and *ξ*_*k*_^21^:

4 $$\begin{array}{rcl}{V}_{k} & = & \langle {\xi }_{k}^{(\sigma )}|{E}^{(\sigma )}\rangle \\  & = & {\rho }_{k}^{0}\sum _{\sigma }{\xi }_{k}^{(\sigma )}{E}^{(\sigma )}\\  & = & {\rho }_{k}^{0}\sum _{{\sigma }_{k}}[{\xi }_{k}^{({\sigma }_{k})}\langle \sum _{{\sigma }_{N-k}}{E}^{({\sigma }_{k};{\sigma }_{N-k})}\rangle ]\mathrm{.}\end{array}$$

Here 〈|〉 denotes inner product for configuration space, ${\rho }_{k}^{0}$ denotes a normalized constant for the inner product, and the term in 〈〉 denotes over all possible configurations except for pair *k* (i.e., *σ*_*N*−*k*_). Transformation from the second to the third line means that summation is taken in terms of possible configurations on pair *k* (i.e., *σ*_*k*_) instead of a summation over all possible configurations of *σ*. This transformation explicitly shows us that when a given system includes many-body interactions $\langle {\sum }_{{\sigma }_{N-k}}{E}^{({\sigma }_{k};{\sigma }_{N-k})}\rangle$ should include the many-body interactions except for that of *k*. Therefore, even though in this study pair-wise *k*s are only considered, our method correctly treats essential many-body interactions.

### Short-range-order in our simulation

We calculated the SROs of clusters using the Metropolis algorithm with a 100 × 100 two-dimensional triangular lattice under fixed composition. Note that, to explicitly consider the size of the nano-sized clusters without loss of validity, interactions of 1–3NN pairs are configured as having a very high so as not to overlap with each other.

### Determination of many-body interactions from a single STM image

We first assume that the measured structure obtained by STM is in thermodynamic equilibrium. Then, the expectation value of the *k*th coordination of the structure at temperature *T*, *ξ*_*k*_(*T*), can be given in the framework of classical statistical mechanics as

5 $${\xi }_{k}(T)={Z}^{-1}\sum _{\sigma }{\xi }_{k}^{(\sigma )}\exp (-\frac{{E}^{(\sigma )}}{{k}_{{\rm{B}}}T}),$$

where summation is taken over all possible configurations *σ*, and *Z* denotes a partition function.

Under these conditions, we have recently derived the relationship^16,17^ between the structure, $${\bf{Q}}(T)=$$$$\{\langle {\xi }_{1}\rangle (T),\,\cdot \,\cdot \,\cdot \,,\langle {\xi }_{f}\rangle (T)\}$$ and the energy coefficients in Eq. (4), **V** = {*V*_1_, …, *V*_*f*_}, in an explicit matrix form:

6 $$\begin{array}{c}{{\bf{Q}}}_{{\rm{ave}}}+{\rm{\Gamma }}(T)\cdot {\bf{V}}\simeq {\bf{Q}}(T)\\ {{\rm{\Gamma }}}_{ik}(T)=-\frac{1}{{k}_{{\rm{B}}}T}{S}_{ik},\end{array}$$

where Q_ave_ represents a linearly averaged configuration and *S*_*ik*_ denotes an element of the covariance matrix for the configurational density of states in $({\xi }_{i},{\xi }_{k})$ two-dimensional space for a non-interacting system. Both of these quantities can therefore be known *a priori* without any information about energy or temperature. From Eq. (6), we can thus directly determine the energy coefficients defined in Eq. (4) from a measured structure using the equation

7 $${\bf{V}}\simeq {{\rm{\Gamma }}}^{-1}\cdot ({\bf{Q}}(T)-{{\bf{Q}}}_{{\rm{ave}}}).$$

### Constructing the interface phase diagram

Recently, we proposed a new method^22^ based on the Wang-Landau algorithm to construct an alloy phase diagram. Since the Wang-Landau algorithm can accurately estimate phases that conventional Monte Carlo methods overlook, this method is suitable for estimating the unknown interface phase diagram.

In this study, the interface is regarded as a Mg–cluster pseudo-binary alloy. To construct the alloy phase diagram, a semi-grand-canonical (SGC) ensemble is more often used than grand-canonical or canonical ensemble. In an SGC ensemble, compositions can vary under the number of atoms, *N*, kept fixed, i.e., we handle the difference of chemical potentials for each constituent element, $\mu ={\mu }_{{\rm{cluster}}}-{\mu }_{{\rm{Mg}}}$ in spite of handling compositions. The total energy, *E*_SGC_, is defined as ${E}_{{\rm{S}}{\rm{G}}{\rm{C}}}=E-\mu Nx$, and the free energy, $\varphi$, is defined as $\varphi =-\,{k}_{{\rm{B}}}T\,\mathrm{ln}\,Y$, with Boltzmann constant *k*_B_ and the partition function in the SGC ensemble *Y* defined as:

8 $$Y(T,\mu )=\sum _{{E}_{{\rm{SGC}}}}W({E}_{{\rm{SGC}}})\exp (-\frac{{E}_{{\rm{SGC}}}}{{k}_{{\rm{B}}}T}),$$

where *W* denotes density of states (DOS). Note that Helmholtz free energy *F* in the canonical ensemble is related to the free energy $\varphi$ in the SGC ensemble through Legendre transformation:

9 $$\varphi =F-\mu Nx.$$

We can obtain *x* using partial differentiation by interpolating $\varphi$ for each chemical potential:

10 $$x=-\frac{\partial \varphi }{\partial (\mu N)}.$$

The above procedure compensates for a disadvantage of the Wang-Landau algorithm, which cannot not directly calculate a thermodynamically averaged value unlike the Metropolis algorithm.

We estimated the DOS for a 30×30 two-dimensional triangular lattice with single-spin-flip update so as to prevent nanoclusters from overlapping with each other. Using the DOS, Eqs (8) and (10), we obtained the free energy *F* in the canonical ensemble and estimated its phase diagram.

## References

[1] Lin S-k, Yeh C-k, Xie W, Liu Y-c, Yoshimura M (2013). *Ab initio*-aided CALPHAD thermodynamic modeling of the Sn-Pb binary system under current stressing. Scientific Reports.

[2] Turchi PEA (2005). Application of ab initio and CALPHAD thermodynamics to Mo-Ta-W alloys. Phys. Rev. B.

[3] Kresse G, Joubert D (1999). From ultrasoft pseudopotentials to the projector augmented-wave method. Phys. Rev. B.

[4] Kresse G, Furthmüller J (1996). Efficient iterative schemes for *ab initio* total-energy calculations using a plane-wave basis set. Phys. Rev. B.

[5] Kresse G, Hafner J (1993). *Ab initio* molecular dynamics for liquid metals. Phys. Rev. B.

[6] van de Walle A, Asta M, Ceder G (2002). The alloy theoretic automated toolkit: A user guide. Calphad.

[7] van de Walle A, Ceder G (2002). Automating first-principles phase diagram calculations. Journal of Phase Equilibria.

[8] Puchala B, Van der Ven A (2013). Thermodynamics of the Zr-O system from first-principles calculations. Phys. Rev. B.

[9] Ruban AV, Abrikosov IA (2008). Configurational thermodynamics of alloys from first principles: effective cluster interactions. Reports on Progress in Physics.

[10] Van der Ven A, Ceder G (2005). Vacancies in ordered and disordered binary alloys treated with the cluster expansion. Phys. Rev. B.

[11] Zhu Y, Morton A, Nie J (2010). The 18 R and 14 H long-period stacking ordered structures in Mg-Y-Zn alloys. Acta Materialia.

[12] Xu C (2012). Ultra high-strength Mg-Gd-Y-Zn-Zr alloy sheets processed by large-strain hot rolling and ageing. Materials Science and Engineering: A.

[13] Xu C (2017). Effect of LPSO and SFs on microstructure evolution and mechanical properties of Mg-Gd-Y-Zn-Zr alloy. Scientific Reports.

[14] Kimizuka H, Kurokawa S, Yamaguchi A, Sakai A, Ogata S (2014). Two-Dimensional Ordering of Solute Nanoclusters at a Close-Packed Stacking Fault: Modeling and Experimental Analysis. Scientific Reports.

[15] Minamoto S, Horiuchi T, Miura S (2015). Refinement of Thermodynamic Parameters of the Mg_24_ Y_5_, W and H Phases in the Mg-Zn-Y System. MATERIALS TRANSACTIONS.

[16] Yuge K (2016). Equilibrium Macroscopic Structure Revisited from Spatial Constraint. Journal of the Physical Society of Japan.

[17] Yuge, K. *Geometric Nature Rules Structure/Potential-Energy Correspondence*. Preprint at https://arxiv.org/abs/1704.07725 (2017).

[18] Kurokawa S, Yamaguchi A, Sakai A (2013). An Attempt to Image Chemical Ordering in Close-Packed Layer of Mg-Zn-Y 18 R Long-Period Stacking-Ordered Structure by Scanning Tunneling Microscopy. MATERIALS TRANSACTIONS.

[19] Kyosuke K, Kaito N, Akihide M, Akira Y, Haruyuki I (2015). Crystal structures of highly-ordered long-period stacking-ordered phases with 18 R, 14 H and 10H-type stacking sequences in the Mg-Zn-Y system. Acta Materialia.

[20] Sanchez JM, Ducastelle F, Gratias D (1984). Generalized cluster description of multicomponent systems. Physica A: Statistical Mechanics and its Applications.

[21] Fontaine, D. D. Cluster Approach to Order-Disorder Transformations in Alloys. vol. 47 of *Solid State Physics*, 33–176 (Academic Press, 1994).

[22] Takeuchi K, Tanaka R, Yuge K (2017). New Wang-Landau approach to obtain phase diagrams for multicomponent alloys. Phys. Rev. B.

